# Exchange of single amino acids at different positions of a recombinant protein affects metabolic burden in *Escherichia coli*

**DOI:** 10.1186/s12934-015-0191-y

**Published:** 2015-01-23

**Authors:** Natalie Rahmen, Alexander Fulton, Nina Ihling, Marzio Magni, Karl-Erich Jaeger, Jochen Büchs

**Affiliations:** AVT – Biochemical Engineering, RWTH Aachen University, Worringerweg 1, D-52074 Aachen, Germany; Institute for Molecular Enzyme Technology, Heinrich-Heine-University Düsseldorf, Forschungszentrum Jülich, D-52426, Jülich, Germany; Institute of Bio- and Geosciences IBG-1: Biotechnology, Forschungszentrum Jülich GmbH, D-52426 Jülich, Germany

**Keywords:** Recombinant protein, Metabolic burden, *Escherichia coli*, *Bacillus subtilis* lipase A (BSLA), Amino acid exchange, Metabolic activity, Online monitoring, Oxygen Transfer Rate (OTR), Respiration Activity MOnitoring System (RAMOS), BioLector

## Abstract

**Background:**

*Escherichia coli* is commonly used in academia and industry for expressing recombinant proteins because of its well-characterized molecular genetics and the availability of numerous expression vectors and strains. One important issue during recombinant protein production is the so-called ‘metabolic burden’: the material and energy normally reserved for microbial metabolism which is sapped from the bacterium to produce the recombinant protein. This material and energy drain harms biomass formation and modifies respiration. To the best of our knowledge, no research has investigated so far whether a single amino acid exchange in a recombinant protein affects the metabolic burden phenomenon. Thus, in this study, 15 *E. coli* BL21(DE3) clones expressing either the fusion tags, a recombinant wild type lipase, or 13 different lipase variants are investigated to quantitatively analyze the respective effects of single amino acid exchanges at different positions on respiration, biomass and protein production of each clone. Therefore, two small-scale online monitoring systems, namely a Respiration Activity MOnitoring System (RAMOS) and a microtiter plate based cultivation system (BioLector) are applied.

**Results:**

Upon expression of all enzyme variants, strong variations were found in the Oxygen Transfer Rate (OTR), biomass and protein (lipase) production of the respective *E. coli* clones. Two distinct patterns of respiration behavior were observed and, so, the clones could be classified into two groups (Type A and B). Potential factors to explain these patterns were evaluated (e.g. plasmid copy number, inclusion body formation). However, no decisive factor could yet be identified. Five distinct cultivation phases could be determined from OTR curves which give real-time information about carbon source consumption, biomass and protein production. In general, it was found that the quantity of product increased with the duration of active respiration.

**Conclusions:**

This work demonstrates that single amino acid exchanges in a recombinant protein influence the metabolic burden during protein production. The small-scale online monitoring devices RAMOS and BioLector enable the real-time detection of even smallest differences in respiration behavior, biomass and protein production in the *E. coli* clones investigated. Hence, this study underscores the importance of parallel online monitoring systems to unveil the relevance of single amino acid exchanges for the recombinant protein production.

**Electronic supplementary material:**

The online version of this article (doi:10.1186/s12934-015-0191-y) contains supplementary material, which is available to authorized users.

## Background

Among many available microbial systems, *Escherichia coli* is the most commonly used prokaryotic expression system for the production of recombinant proteins. This is due to its well-known genetics, its ability to grow rapidly to high cell densities on inexpensive mineral media, as well as the large number of available cloning vectors and optimized host strains [1,2].

A frequently used *E. coli*-based expression system is the bacteriophage T7 RNA polymerase-controlled system [3] under control of the *lac* operon. Induction with isopropyl β-D-thiogalactopyranoside (IPTG) requires the manual addition of the inducer as well as biomass monitoring to determine the optimal induction time point. By contrast, autoinduction is controlled by the metabolism of the expression host, i.e. *E. coli*, and thus is distinguished by cell growth and subsequent product formation [4-6]. To achieve this, a carbon source mixture of glucose, glycerol, and lactose is commonly used. During the initial growth phase, glucose is preferentially consumed and protein formation is suppressed by catabolite repression [7]. Once glucose is depleted, lactose and glycerol are taken up and consumed. Lactose is partially converted into allolactose, which acts as the physiological inducer of the *lac* operon [5]. Glycerol serves as additional energy source. Several modified complex autoinduction media are based on the composition described by Studier [4]. Commercial sources of such media are also available, but are often expensive and have an undefined composition [6]. Mineral autoinduction media have a defined chemical composition which allows a better understanding of metabolic processes during induction and protein expression.

The production of recombinant proteins is one of the most energy and raw material consuming processes. It activates stress responses and causes significant alterations in host cell metabolism. ‘Metabolic burden’ is defined as the draining of raw materials and energy from the physiological microbial metabolism as the result of this protein production [8-11]. Many reviews have summarized the main challenges in recombinant protein production and potential factors influencing the metabolic burden, as well as strategies to overcome metabolic burden and optimize protein production [12-17]. One of the most prevalently observed changes in host-cell physiology due to metabolic burden is a decrease in growth rate which can be impaired in different ways [18,19]. The general influence of the amino acid sequence of recombinant proteins on the metabolic burden phenomenon has already been evaluated in some studies [20,21]. Palmen *et al.* found that slight differences in the amino acid composition of a cofactor-dependent enzyme significantly affect the expression and cultivation progress. They correlated the binding strength of the cofactor thiamin diphosphate of the recombinant benzoylformate decarboxylase to the differences in the metabolic activity [20]. Furthermore, the metabolic costs of the amino acid biosynthesis in *E. coli* were calculated as high-energy phosphate bonds contained in ATP and GTP molecules, and according to available hydrogen atoms in NADH, NADPH, and FADH_2_ molecules, respectively [22,23]. Tryptophan (W), phenylalanine (F), tyrosine (Y), histidine (H) and methionine (M) were identified as amino acids leading to the highest energetic costs during their biosynthesis [22]. For the cellular stress caused by the recombinant protein production, Bonomo and Gill also discovered that the amino acid sequence itself plays an important role [21]. Using the same host organism and expression system, they investigated the growth behavior upon expression of two different polypeptides. The first polypeptide was composed of the most rarely used amino acids causing the highest energetic costs, whereas the second one was composed of the most abundant and, thus, energetically inexpensive amino acids. The expression of the first polypeptide led to a strong decrease in growth [21] due to a stringent-like response [24-26].

Some approaches to determine and quantify the metabolic burden [27-31] are either based on sampling and subsequent offline monitoring techniques or described for large-scale fermentations requiring considerable equipment, chemicals, and time.

This study aims to investigate the influence of single amino acid exchanges at different positions of a recombinant enzyme on metabolic activity and expression of the host *E. coli* BL21(DE3) using two small-scale online monitoring devices. The first device is the Respiration Activity MOnitoring System (RAMOS) [32,33] which enables the online measurement of the Oxygen Transfer Rate (OTR) as characteristic parameter for the metabolic activity of the bacteria. The second device is the BioLector [34,35] which measures scattered light and fluorescence to trace biomass and protein formation during cultivation. Within this study, 15 *E. coli* BL21(DE3) clones that only differ by the exchange of single amino acids are investigated. Thereby, the amino acid exchanges are distributed over the entire amino acid sequence (Table 1). The target protein is the lipase LipA from *Bacillus subtilis* (BSLA) [36] which is fused to a flavin-based fluorescent protein (FbFP) derived from the Light, Oxygen, Voltage (LOV) domain of the *Bacillus subtilis* YtvA photoreceptor (LOV tag) [37,38]. For cultivation under inducing conditions a mineral autoinduction medium was used.

**Table 1:** **Applied clones and abbreviations**

**Clone**	**Abbreviation**
*E. coli* BL21(DE3):pET22b(+)–His_6_-LOV	His-LOV
*E. coli* BL21(DE3):pET22b(+)–His_6_-LOV-BSLA-wt	Wild type enzyme (His-LOV-BSLA)
*E. coli* BL21(DE3):pET22b(+)–His_6_-LOV-BSLA-Ala1Trp	A1W
*E. coli* BL21(DE3):pET22b(+)–His_6_-LOV-BSLA-His10Asp	H10D
*E. coli* BL21(DE3):pET22b(+)–His_6_-LOV-BSLA-Ile12Cys	I12C
*E. coli* BL21(DE3):pET22b(+)–His_6_-LOV-BSLA-Phe17Pro	F17P
*E. coli* BL21(DE3):pET22b(+)–His_6_-LOV-BSLA-Lys23stop	K23stop
*E. coli* BL21(DE3):pET22b(+)–His_6_-LOV-BSLA-Ser56Pro	S56P
*E. coli* BL21(DE3):pET22b(+)–His_6_-LOV-BSLA-Asp91Arg	D91R
*E. coli* BL21(DE3):pET22b(+)–His_6_-LOV-BSLA-Gly93Tyr	G93Y
*E. coli* BL21(DE3):pET22b(+)–His_6_-LOV-BSLA-Val99Lys	V99K
*E. coli* BL21(DE3):pET22b(+)–His_6_-LOV-BSLA-Leu102Trp	L102W
*E. coli* BL21(DE3):pET22b(+)–His_6_-LOV-BSLA-Ser167Pro	S167P
*E. coli* BL21(DE3):pET22b(+)–His_6_-LOV-BSLA-Lys170Glu	K170E
*E. coli* BL21(DE3):pET22b(+)–His_6_-LOV-BSLA-Gly175Phe	G175F

The investigated *E. coli* BL21(DE3) clones contain plasmid pET22b(+) harboring the gene encoding fusion tags, wild type BSLA or BSLA variants. Each variant contains a single amino acid exchange at a different position chosen randomly and distributed over the entire enzyme sequence of 181 amino acids.

## Results and discussion

Table 1 lists the investigated *E. coli* BL21(DE3) clones. Each clone is named after the particular enzyme variant it expresses. One clone expresses a fusion of N-terminal polyhistidine (His_6_) tag and LOV tag [37,38] (Additional file 1A). The other clones express wild type *Bacillus subtilis* lipase A (BSLA) or BSLA variants fused to His_6_ and LOV tag (Additional file 1B). Each BSLA variant contains a single amino acid exchange at a different position chosen randomly and distributed over the entire enzyme sequence of 181 amino acids. In variant K23stop the natural amino acid at position 23 (lysine) is replaced by a stop codon leading to a truncated protein.

**Figure 1:**
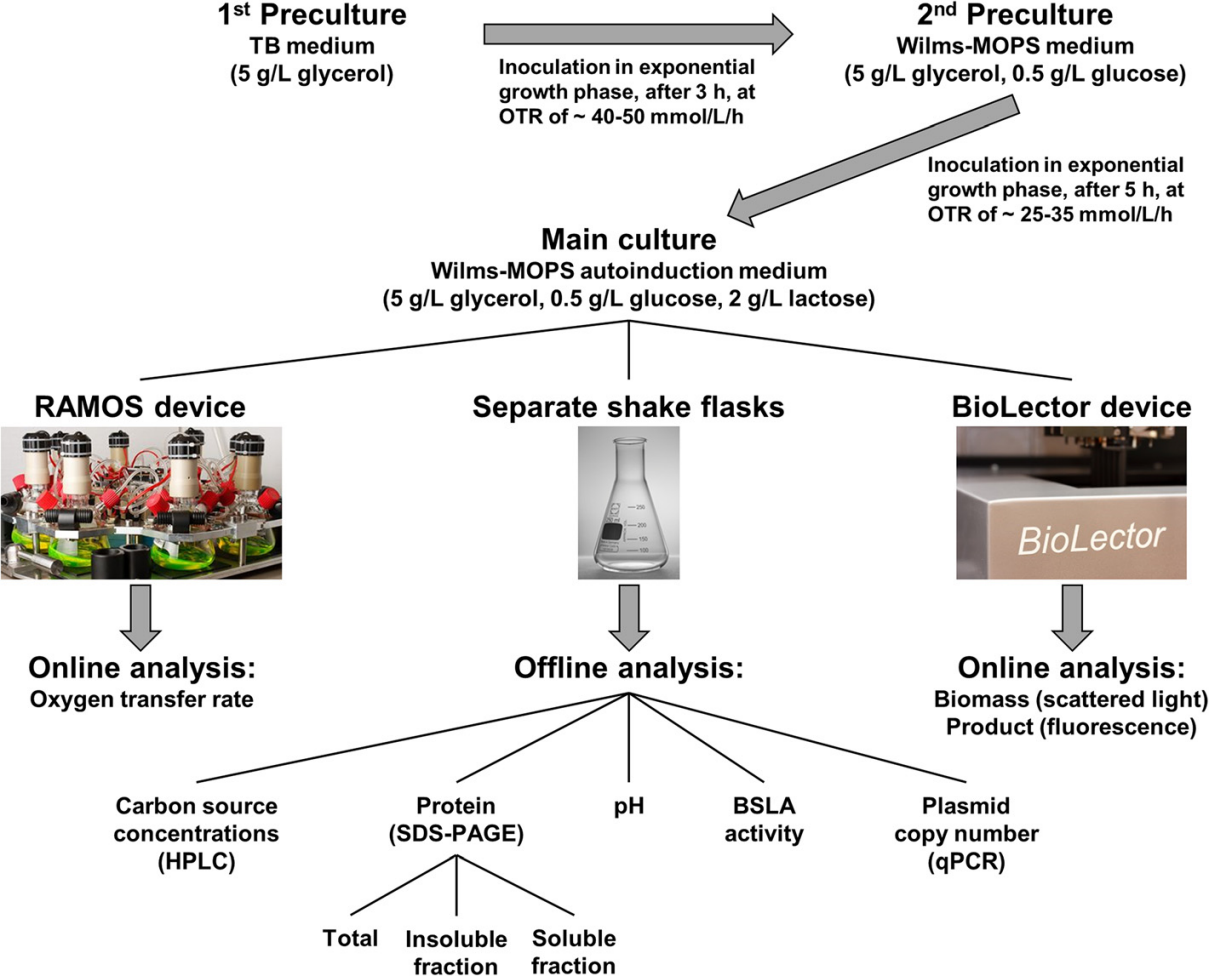
**Overview of different cultivation steps, cultivation systems, and measured online and offline data.** First preculture is performed in complex non-inducing TB medium, second preculture in non-inducing Wilms-MOPS mineral medium. Both precultures are carried out in 250 mL flasks in a RAMOS (Respiration Activity MOnitoring System) device. The main culture is performed in Wilms-MOPS mineral autoinduction medium in three cultivation systems in parallel: RAMOS device (250 mL shake flasks), separate 250 mL shake flasks and 48-well Flowerplate in a BioLector device. The RAMOS device allows an online measurement of the oxygen transfer rate (OTR) of the culture. Separate shake flasks are used for offline analysis. The BioLector device monitors the formation of biomass (scattered light intensity) and product (fluorescence).

An overview of the underlying cultivation procedure, the different applied cultivation systems and the determined online and offline data is given in Figure 1. A detailed description can be found in the Methods section.

### Precultivations under non-inducing conditions

**Figure 2:**
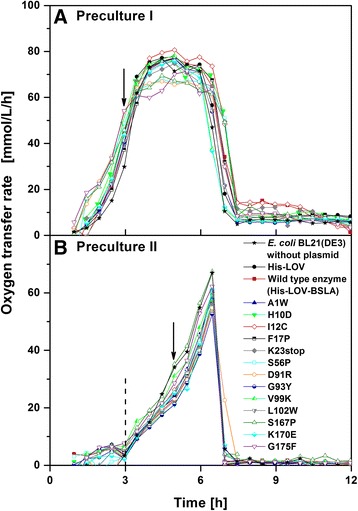
**Precultivations of 15** ***E. coli*** **BL21(DE3) clones and an** ***E. coli*** **BL21(DE3) control without plasmid under non-inducing conditions.** Oxygen transfer rate (OTR) as a function of time for: **(A)** first preculture performed in complex TB medium, and **(B)** second preculture performed in Wilms-MOPS mineral medium. The arrows indicate the time points the cultures were harvested and used for inoculating the next cultivation step. The dotted line in **(B)** highlights the depletion of glucose. Cultivation conditions: 37°C, 250 mL flasks, filling volume 10 mL, shaking frequency 350 rpm, and shaking diameter 50 mm.

The precultivations under non-inducing conditions were consecutively performed in complex and mineral medium, respectively, to compare the respiration behavior and to determine the optimal OTR and time point for the inoculation of the subsequent culture (Figure 2). For the first precultivation, the investigated clones were cultivated in complex TB medium with glycerol as the main carbon source.

As illustrated in Figure 2A, the OTR curves of all clones depict a quite similar respiration behavior. They show the typical growth of *E. coli* in TB medium as described earlier [39] and as also observed in other studies where *E. coli* was cultivated under similar conditions [19,20]. The exponential growth phase exhibits a maximum OTR of 65**–**80 mmol/L/h after 5 h of cultivation. The horizontal plateau indicates a phase of oxygen limitation. Subsequently, the sharp decrease in the OTR to a value of about 10 mmol/L/h indicates the depletion of all original carbon sources. Some residual growth is attributed to the consumption of acetate as overflow metabolite (data not shown) as described in general [40,41] and in particular for TB medium [39]. In further experiments, the first preculture was harvested during its exponential growth phase after 3 h at an OTR of 40**–**50 mmol/L/h (arrow in Figure 2A), and it was further used for the inoculation of the second preculture.

The second preculture was cultivated in modified Wilms-MOPS mineral medium containing 0.5 g/L glucose and 5 g/L glycerol as carbon sources. Figure 2B illustrates that all investigated *E. coli* clones depict the same respiration behavior. After a first initial increase, the OTR slightly decreases after 3 h due to the depletion of the preferred carbon source glucose (dotted line in Figure 2B). Afterwards, exponential growth on glycerol results in a maximum OTR of 55–65 mmol/L/h after 6.5 h. The subsequent plummeting of the OTR to 0 mmol/L/h indicates the exhaustion of all original carbon sources. Due to the defined composition of the cultivation medium, the low carbon source concentration, and the prevention of an oxygen limitation, no acetate formation was observed during the cultivation in Wilms-MOPS mineral medium (data not shown). For subsequent experiments, the second preculture was harvested during its exponential growth phase after 5 h at an OTR of 25–35 mmol/L/h (arrow in Figure 2B), and was used for inoculating the main culture.

The investigated *E. coli* clones bear plasmids with genes encoding BSLA with different single amino acid exchanges. In this study, the plasmids had no effect on *E. coli* metabolism under non-inducing conditions. According to literature, the plasmid pET22b(+) used here usually occurs with about 40 copies per cell [42] thus belonging to the high copy number plasmids. In our study, 25 copies per genome could be measured under non-inducing conditions. In general, increased plasmid copy numbers can provoke decreasing growth rates [43-46]. Nevertheless, in comparison to *E. coli* BL21(DE3) not bearing any plasmid (Figure 2, Additional file 2) no effect on growth and in consequence no negative influence on metabolism were observed for the investigated clones during non-inducing cultivations in this study. This may be explained by the relatively small plasmid size which influences growth to a lesser extent than larger plasmids [47,48]. The small variation in the gene sequences of the studied clones had no influence on *E. coli* respiration behavior under non-inducing conditions. This suggests that the metabolism is not burdened, because an increase in copy number is not induced and the expression system used here is tightly controlled and not leading to any unintended recombinant protein expression as previously described for expression studies using complex media [49,50].

### Cultivation under inducing conditions

#### Influence of single amino acid exchanges and definition of two distinct types of respiration behavior

**Figure 3:**
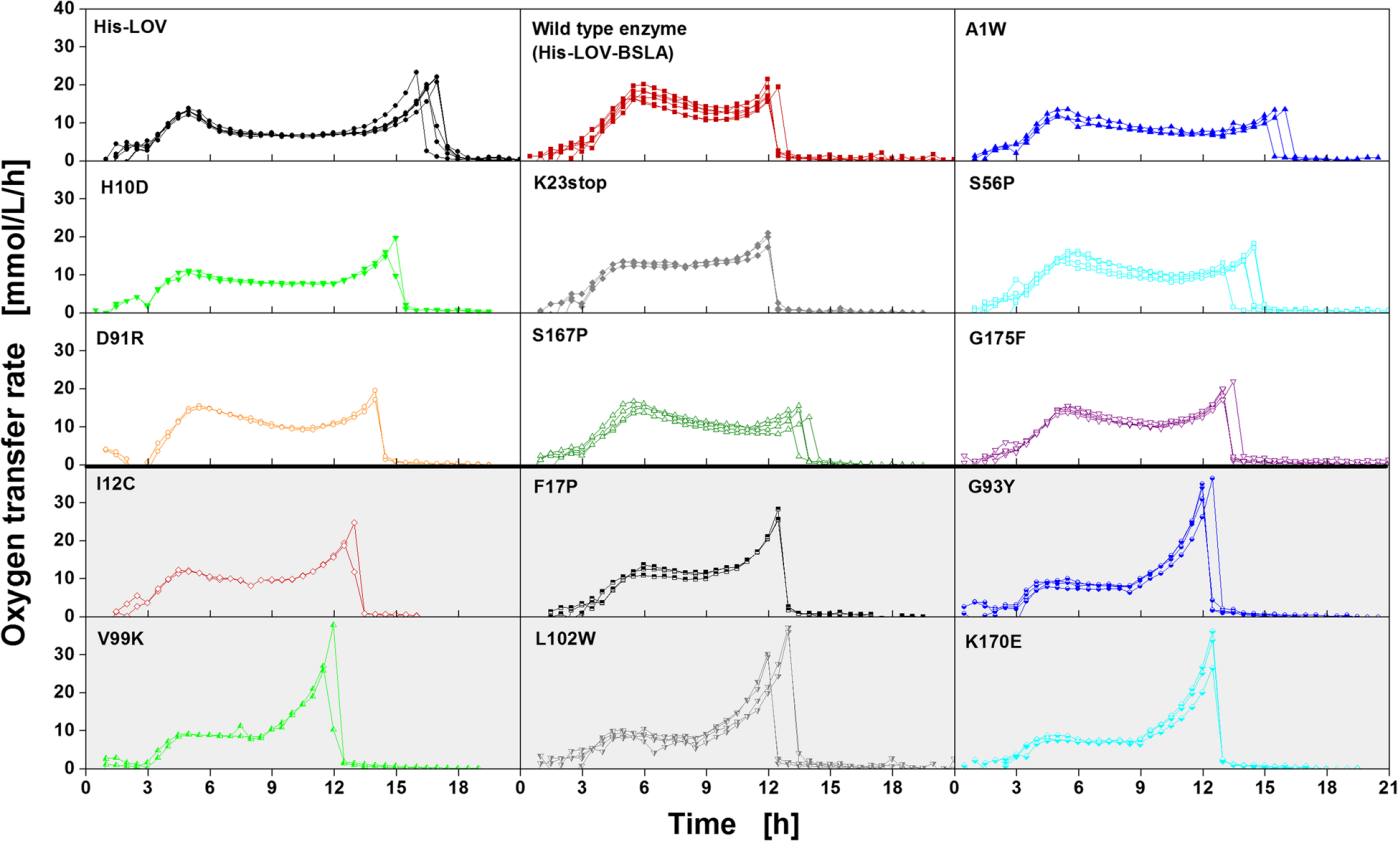
**Reproducibility of main cultivations of 15** ***E. coli*** **BL21(DE3) clones under inducing conditions expressing fusion tags, wild type BSLA, or different BSLA variants as specified in Table** **1** **.** Oxygen transfer rate (OTR) as function of time obtained from 2–6 independent replicate experiments performed in Wilms-MOPS mineral autoinduction medium containing 0.5 g/L glucose, 2 g/L lactose and 5 g/L glycerol. According to their respiration behavior, the clones can be classified into Type A (white background) and Type B (grey background). The nine clones belonging to respiration behavior Type A reach an OTR of 15–20 mmol/L/h in both OTR peaks, whereas the six clones belonging to respiration behavior Type B increase up to only about 10 mmol/L/h in their first OTR peak and reach 25–40 mmol/L/h in the second OTR peak. Cultivation conditions: 37°C, 250 mL flasks, filling volume 10 mL, shaking frequency 350 rpm, and shaking diameter 50 mm.

For the main cultivation under inducing conditions using lactose as inducer, the 15 investigated *E. coli* BL21(DE3) clones were cultivated in Wilms-MOPS mineral autoinduction medium to determine their respiration activity under inducing conditions. Figure 3 depicts the respective OTR curves of all clones as a function of time obtained from 2–6 independent replicate experiments.

Despite a similar respiration behavior of all clones cultivated under non-inducing conditions (Figure 2), Figure 3 shows highly reproducible differences among the clones with respect to respiration under inducing conditions. In contrast, an *E. coli* BL21(DE3) without plasmid delivers the expected identical respiration behavior as under non-inducing conditions (Additional file 2). The differences in respiration presented in Figure 3 resemble the behavior of clones that express different recombinant proteins in complex autoinduction medium investigated by Kunze *et al.* [19]. To allow a better understanding of the metabolic processes during induction and protein expression, in this current study, a mineral autoinduction medium with a defined composition was used. To verify that the obtained differences in respiration (Figure 3) are not caused by the growth medium itself, in one additional experiment all 15 *E. coli* BL21(DE3) clones were cultivated in complex autoinduction medium. Thereby, it could be shown that differences between the clones also arise during cultivation in complex autoinduction medium (Additional file 3). After an exponential increase in OTR within the first 3.5 h, the OTR of some clones (e.g. I12C, K23stop, S56P, G175F) further increases continuously until reaching a maximum. For other clones (e.g. A1W, F17P, G93Y, K170E) the OTR drops after a short constant phase and afterwards increases again. The clone His-LOV even shows a sharper decrease in OTR. After further 1.5 h, another increase to its maximum OTR is observed. The differences in complex autoinduction medium, though, are less pronounced than in mineral autoinduction medium. This can be explained by the fact that under the chosen cultivation conditions (equal to those in Figure 3), the cultures are oxygen limited over a wide range of the cultivation due to the very rich medium composition. In mineral autoinduction medium the differences between the investigated clones appear much clearer. Remarkably, significant differences in OTR occur even though the recombinant target proteins (BSLA) differed in just a single amino acid. These reproducible differences in the metabolic activity (Figure 3), thus, lead to the conclusion that even very small differences in the gene sequence coding for BSLA influence the host organism in quite different ways during cultivation under inducing conditions. Despite the diverse OTR patterns, the clones are classified into two types of respiration behavior (Type A and Type B) according to their maximum OTRs and cultivation durations. Even though there seems to be a continuous change between the respiration behaviors of the investigated clones, in this study the attempt for a classification is made, as this simplifies the discussion about the general differences observed. In former publications of Lee and Ramirez [18] and Kunze *et al.* [19] *E. coli* clones expressing different recombinant proteins were already classified according to growth and respiration. As Figure 3 illustrates, all clones show an initial increase in OTR due to their growth on the preferred carbon source glucose. As already noticed from second precultivations (Figure 2B), after 3 h, the depletion of glucose leads to a small drop in the OTR, followed by a further increase to a first OTR peak after 5–6 h. The nine clones classified into respiration behavior Type A (Figure 3, white background) reach an OTR of 15–20 mmol/L/h, whereas the OTRs of the six clones belonging to respiration behavior Type B (Figure 3, grey background) increase up to only about 10 mmol/L/h. After a phase of decreasing or constant OTR of various lengths, all clones show an OTR increase until a second peak is reached at 15–20 mmol/L/h (Type A) or 25–40 mmol/L/h (Type B), respectively. The end of the cultivation is indicated by the sharp drop in OTR. The cultivation takes 12–17 h (Type A) or 12–13 h (Type B), respectively.

**Figure 4:**
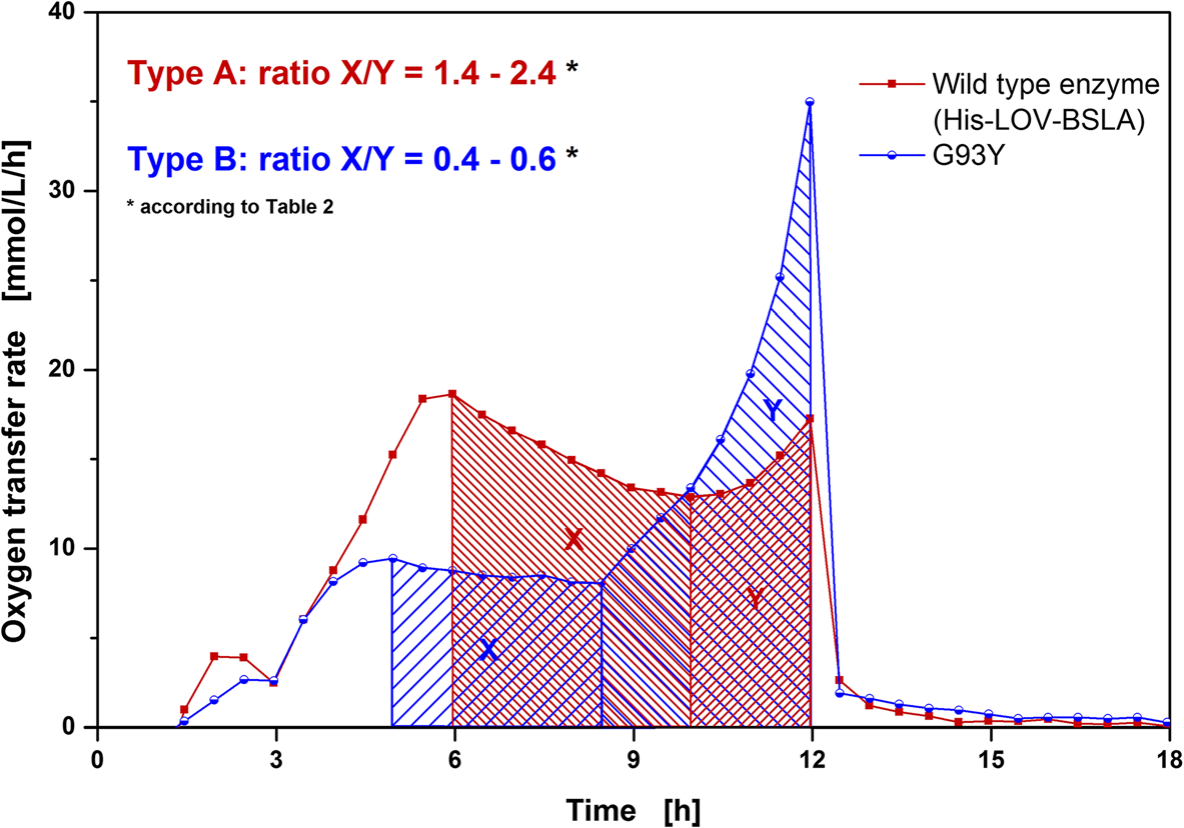
**Quantitative classification of 15** ***E. coli*** **BL21(DE3) clones into Type A and Type B according to their respiration behavior.** Comparison of the respiration behavior (oxygen transfer rate) as function of time of Type A and Type B clones calculating the ratio between integral X (from first OTR peak to minimum) and integral Y (from minimum to second peak). Integral X represents the phase of protein formation; integral Y represents the second growth phase on residual glycerol. According to Table 2, the ratio X/Y is in the range of 1.4-2.4 for the nine clones specified in Figure 3 belonging to Type A, and in a range of 0.4-0.6 for the six clones belonging to Type B.

Besides the qualitative classification of the clones into two types of respiration behavior according to their OTR pattern, it was possible to quantitatively classify Type A and Type B. As illustrated in Figure 4, the ratio between integral X (from the first OTR peak to the local minimum) and integral Y (from the minimum to the second peak) was calculated for all individual cultivations presented in Figure 3. As shown later, the integral X represents the phase of protein formation and Y the second growth phase on residual glycerol after lactose is depleted. The mean X/Y ratios of all investigated clones are presented in Table 2. The X/Y ratio of the nine clones representing respiration behavior Type A is in the range of 1.4-2.4, and in the range of 0.4-0.6 for the six clones belonging to respiration behavior Type B. The standard deviation varies between 0.4-7.9%. To allow an easier classification of clones into Type A or Type B respiration behavior according to their X/Y ratio, a critical X/Y ratio is introduced (Table 2). All clones exhibiting a ratio greater than 1.2 can be categorized into Type A respiration behavior, whereas clones with a ratio smaller than 1.2 can be classified into Type B. This critical X/Y ratio offers an easy way to distinguish between Type A and Type B clones in future cultivations.

**Table 2:** **X/Y ratio of Type A and Type B clones**

**Type A clone**	**Arithmetic mean X/Y ratio**	**Standard deviation [%]**	**Type B clone**	**Arithmetic mean X/Y ratio**	**Standard deviation [%]**
**His-LOV**	1.7	7.1	**I12C**	0.6	0.5
**Wild type enzyme (His-LOV-BSLA)**	2.2	4.6	**F17P**	0.6	3.6
**A1W**	2.2	7.9	**G93Y**	0.5	6.7
**H10D**	1.4	2.0	**V99K**	0.5	0.4
**K23stop**	1.5	2.9	**L102W**	0.4	4.5
**S56P**	1.9	3.8	**K170E**	0.4	3.3
**D91R**	1.4	2.5			
**S167P**	2.4	7.5			
**G175F**	1.4	1.8			
**Type A**	**1.4 – 2.4**		**Type B**	**0.4 – 0.6**	
Type A*	**>1.2**		Type B*	**<1.2**	

Ratio between integral X (representing protein production phase) and integral Y (representing second growth phase) determined for the 15 investigated *E. coli* BL21(DE3) clones according to Figure 4. For each clone, the X/Y ratio was calculated for all individual cultivations presented in Figure 3. The arithmetic mean as well as the standard deviation (in %) are presented.

*Critical X/Y ratio for simplified classification between Type A and Type B clones.

In the following, some first ideas about possible factors causing the different behavior of Type A and Type B clones will be examined. Preliminary data concerning metabolic costs for the amino acid biosynthesis, enzyme activity, plasmid copy number, formation of inclusion bodies as well as the ratio of insoluble to soluble protein will be presented and discussed. Thereby, our investigations should give a first hint at possible influencing factors without yet studying all parameter in detail.

**Figure 5:**
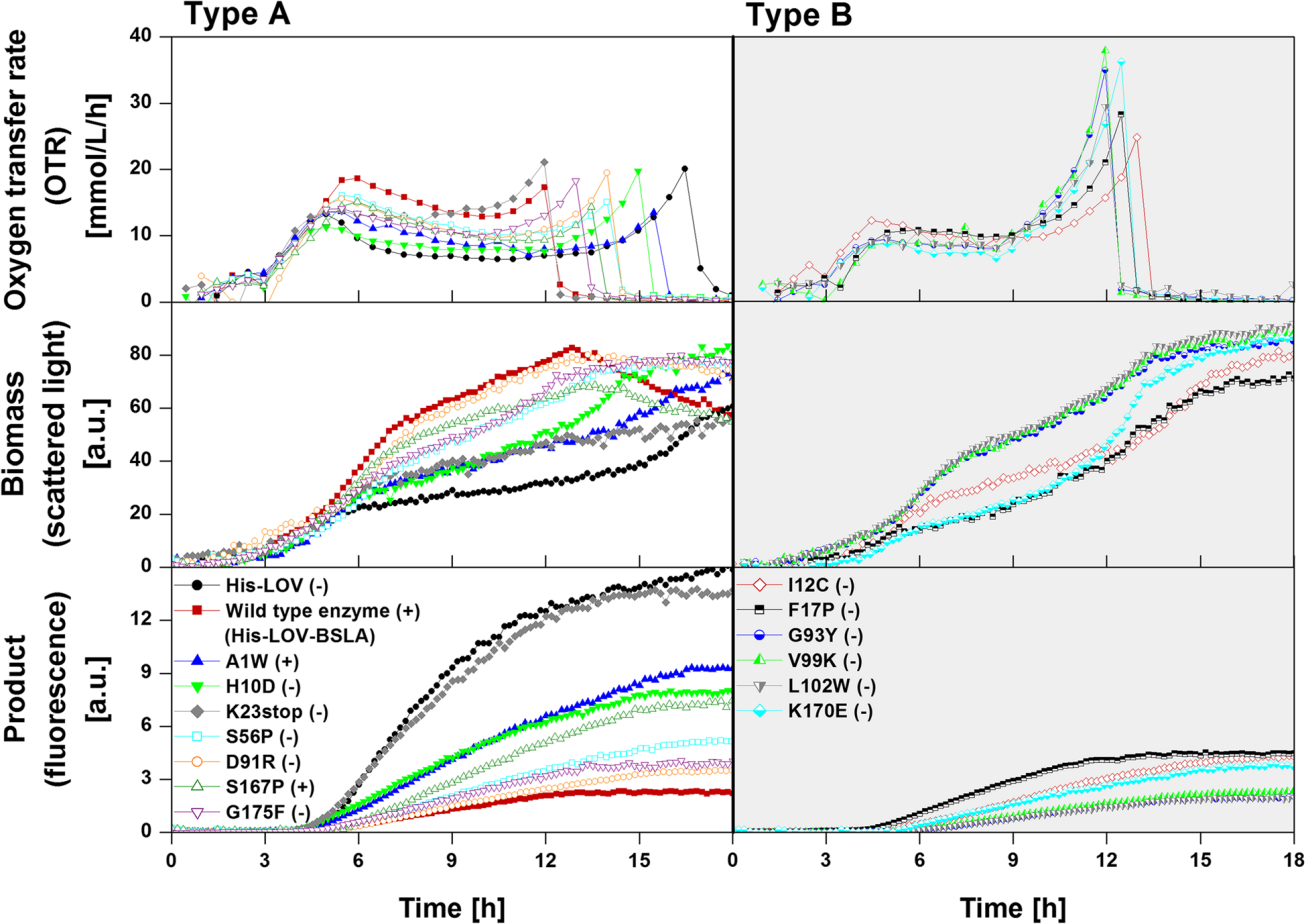
**Comparison of 15** ***E. coli*** **BL21(DE3) clones belonging to respiration behavior Type A and Type B cultivated under inducing conditions.** Oxygen transfer rate (top), biomass (middle) and product formation (bottom) as function of time for *E. coli* BL21(DE3) clones cultivated in Wilms-MOPS mineral autoinduction medium (Type A: white background; Type B: grey background). Clones expressing active *Bacillus subtilis* lipase A (BSLA) are indicated with (+); clones expressing inactive BSLA are indicated with (−). Cultivation conditions: 37°C, 250 mL flasks, filling volume 10 mL, shaking frequency 350 rpm, shaking diameter 50 mm (in RAMOS); 37°C, 48-well Flowerplate, filling volume 1 mL, shaking frequency 1500 rpm, shaking diameter 3 mm (in BioLector).

In addition to an identical host organism and expression system, all *E. coli* BL21(DE3) clones, except His-LOV and K23stop, express identical target proteins, that only differ in one single amino acid within the BSLA sequence (Table 1, Additional file 1). Whether the investigated clones express active (+) or inactive (−) BSLA can be recognized from the figure legend of Figure 5. In contrast to the example given by Palmen *et al.* [20], no cofactor is necessary here for the target protein. Moreover, differences in a stringent-like response [24-26] could be predominantly prevented due to the exchange of just one amino acid. Nevertheless, reproducible differences in growth were observed and a classification into two types of respiration behavior was possible.

To evaluate if increased or decreased energetic costs in amino acid biosynthesis cause the two different types of respiration behavior (Type A and Type B), all amino acid exchanges were examined separately according to Akashi and Gojobori [22]. In Type A as well as in Type B clones, amino acids causing high energetic costs were replaced by amino acids causing lower costs (e.g. H10D for Type A, and F17P for Type B) and inexpensive amino acids were replaced by expensive ones (e.g. A1W for Type A, and L102W for Type B). Hence, metabolic costs of the amino acid biosynthesis could be excluded as a reason for the two presented types of respiration behavior. The change in respiration activity of the different clones caused by a lack of a certain amino acid is also precluded e.g. by the clones S56P and F17P. In both cases, the wild type amino acid has been substituted by proline (P), resulting in an equally increased proline demand. Nevertheless, these two clones belong to different types of respiration behavior. Thus, a lack of proline cannot explain the different OTR patterns.

#### Comparison of respiration behavior Types A and B, and correlations between OTR, biomass and product formation

The differences between Type A and Type B clones were further examined by determination of biomass (*via* scattered light) and product formation (*via* fluorescence) during cultivation in Wilms-MOPS mineral autoinduction medium. A comparison of OTR (using RAMOS), biomass and product formation (using BioLector) of the investigated clones is shown in Figure 5 (Type A: white background; Type B: grey background). In this way, two small-scale online monitoring systems are combined with one another. The reproducibility of biomass and product formation with presentation of measured data points, arithmetic mean and standard deviation is depicted in Additional files 4 and 5, respectively.

As shown in Figure 5, the biomass signal (scattered light intensity) of Type A and B clones increases exponentially until 5–7 h of cultivation, followed by a further linear increase. The time point of the transition from exponential to linear growth roughly correlates with the first OTR peak. Similarly, the duration of the linear increase correlates with the phase of decreasing or constant OTR. At the transition point from exponential to linear growth, clones from Type A attain a biomass signal (scattered light intensity) of 20–40 a.u., while Type B clones show lower biomass signals (scattered light intensities) from 10–30 a.u. This is in good agreement with the height of the first OTR peak, which is 15–20 mmol/L/h for Type A clones, and 10 mmol/L/h for Type B clones. With the final increase in OTR, most clones exhibit another significant increase in the biomass formation. A slight decrease of biomass formation at the end of the cultivation is only observed for a few clones and can be attributed to morphological changes of the cells [51-53]. The final biomass signal (scattered light intensity) for Type A clones ranges from 50–80 a.u., and for Type B clones ranges from 70–90 a.u. Hence, Type B clones showing a strong OTR increase in the second half of cultivation obtain higher final biomass signals.

As also shown in Figure 5, the product formation of all investigated clones starts after 5–6 h, correlating perfectly with the first OTR peak and with the transition from exponential to linear biomass increase. Strong product formation is obtained during the phase of decreasing or constant OTR and linear biomass increase. With the final increase in OTR and biomass signal (scattered light intensity) the product formation rate decreases. The final product fluorescence signal of Type B clones ranges from 1–4.5 a.u., whereas Type A clones produce generally higher amounts of between 2–9 a.u. It has to be considered that the two clones His-LOV and K23stop showing the highest product fluorescence signals (15 a.u. and 12 a.u., respectively) produce shorter target proteins than all other clones (Table 1, Additional file 1) and, therefore, have to be regarded as reference clones. Generally, clones belonging to respiration behavior Type A produce higher amounts of target protein than Type B clones. Type A clones with higher product formation usually result in lower biomass signals. On the other hand, Type B clones showing a strong OTR increase in the second half of the cultivation and a higher biomass formation produce less target protein. Thus, our results agree with the inverse correlation of product formation and growth that was already reported before [54,55]. In agreement with Kunze *et al.* [19], a correlation between the progress of the OTR as function of time and the product formation could be observed. While phases of decreasing or constant OTR resulted from high product formation, an exponential increase in OTR indicated undisturbed cell growth.

The figure legend of Figure 5 also indicates whether the investigated clones express active (+) or inactive (−) BSLA. Twelve out of fifteen clones expressed inactive BSLA. A correlation between respiration behavior and enzyme activity could not be found. Clones producing inactive enzymes were found in both, Type A and Type B, groups, whereby in Type B only inactive enzyme was expressed. A correlation between expressed protein and functionality cannot be found, either. From full-length products, the active variants A1W and S167P are produced in relatively high amounts (9 a.u. and 6 a.u., respectively), whereas the active wild type enzyme is produced in a very low amount (2 a.u.). Furthermore, the variant H10D is produced in a relatively high amount (8 a.u.) even though the expressed protein is inactive.

**Figure 6:**
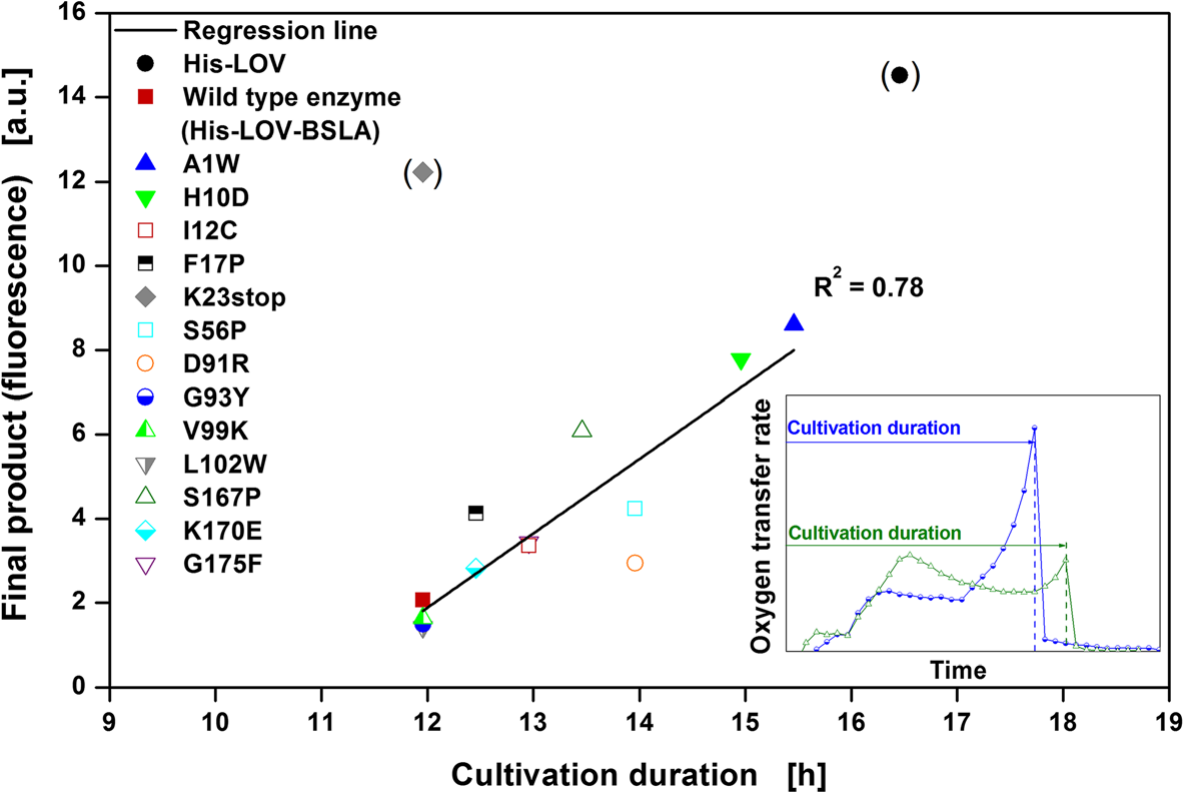
**Correlation of final product fluorescence signal and cultivation duration.** Final product fluorescence signal of 15 *E. coli* BL21(DE3) clones as function of the cultivation duration (until the last peak of the oxygen transfer rate, as shown in the insert). Calculated regression line (R^2^ = 0.78) is based on all clones expressing a full-length target protein as specified in Table 1 and Additional file 1. Shorter products (His-LOV and K23stop) are not considered for the calculation and are marked in parentheses.

For a quantitative analysis of the relation between respiration activity and product formation, Figure 6 shows a correlation of the final product fluorescence signals and the cultivation duration (duration of active respiration). The insert illustrates how the cultivation duration of the particular *E. coli* clones (until the last peak of the OTR) was determined. For the calculation of the regression line, all clones expressing a full-length product as specified in Table 1 and Figure 1 were taken into account. The two clones expressing shorter products (His-LOV and K23stop) were not considered and, therefore, were marked in parentheses in the graph. Even though the investigated clones belong to two different types of respiration behavior, a correlation between the final product fluorescence signals and the cultivation duration was found (R^2^ = 0.78). Longer cultivation durations lead to an enhanced product formation. This agrees with the fact that growth is reduced in case of enhanced product formation, and that with equal amounts of available carbon and energy sources, slower growth results in prolonged cultivation durations.

#### Characterization of Type A and B clones, and definition of five characteristic cultivation phases

**Figure 7:**
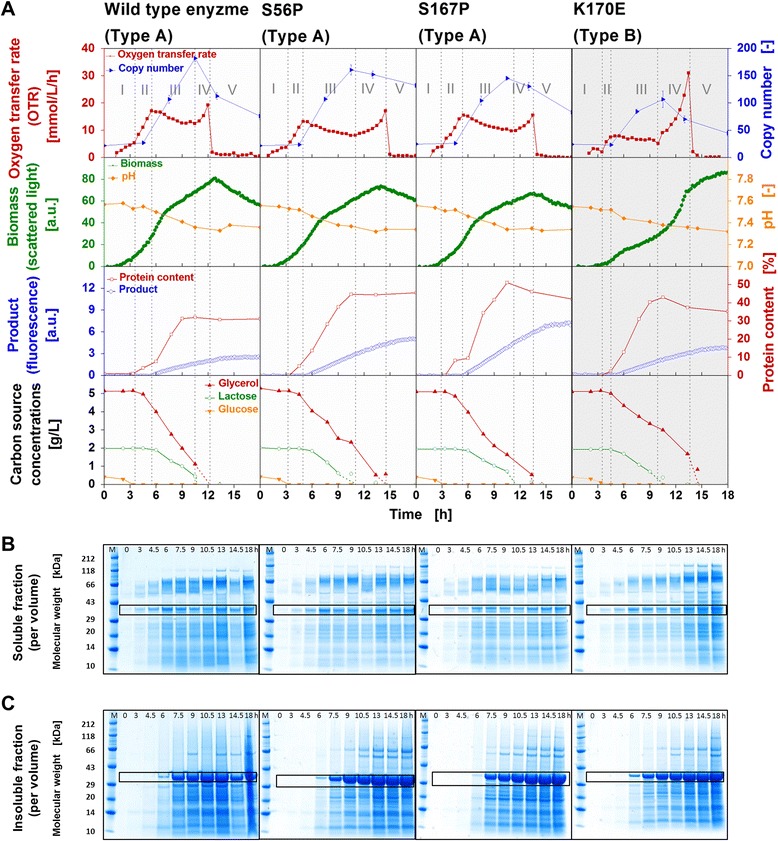
**Detailed characterization of four** ***E. coli*** **BL21(DE3) clones belonging to respiration behavior Type A and Type B cultivated under inducing conditions.** Characterization of four *E. coli* BL21(DE3) clones belonging to respiration behavior Type A (expressing wild type BSLA and variants S56P and S167P) and respiration behavior Type B (BSLA variant K170E) during the cultivation in Wilms-MOPS mineral autoinduction medium. **(A)** Characteristic growth parameters: oxygen transfer rate (OTR), biomass and product formation, carbon source concentrations, copy number of plasmid per genome, pH-value, and protein content of the target protein per total protein of the cell. Type A: white background; Type B: grey background. The five cultivation phases (I-V) are identified by the OTR curves and are separated by the vertical grey dotted lines. (I) increase in OTR due to growth on glucose, the depletion of glucose corresponds with a small OTR drop (first grey dotted line); (II) increase in OTR to first peak (second grey dotted line) due to growth on glycerol; (III) slightly decreasing or constant OTR, respectively, due to product formation on lactose with parallel consumption of glycerol, the depletion of lactose roughly corresponds with the minimum of the OTR (third grey dotted line); (IV) increase in OTR to second peak (forth grey dotted line) due to growth on residual glycerol; (V) end of cultivation, the depletion of glycerol roughly corresponds with the sharp drop in OTR. **(B)** SDS-PAGE analysis showing soluble protein per sample volume, and **(C)** SDS-PAGE analysis showing insoluble protein per sample volume after 0, 3, 4.5, 6, 7.5, 9, 10.5, 13, 14.5, and 18 h of cultivation (target protein band is framed, M = protein marker). Cultivation conditions: 37°C, 250 mL flasks, filling volume 10 mL, shaking frequency 350 rpm, shaking diameter 50 mm (in RAMOS); 37°C, 48-well Flowerplate, filling volume 1 mL, shaking frequency 1500 rpm, shaking diameter 3 mm (in BioLector).

Fermentations were further analyzed by an additional cultivation performed in Wilms-MOPS mineral autoinduction medium with parallel offline analysis (Figure 7). In order to measure the carbon source concentrations, the plasmid copy number related to the number of genome copies, the pH-value, and the target protein content, samples were taken from conventional Erlenmeyer flasks. The measurements were performed for four clones as specified in Figure 7A as examples. Three of those four clones belong to the respiration behavior Type A (white background), while one belongs to the respiration behavior Type B (grey background). Besides the clone expressing the wild type enzyme which is regarded as reference clone, the Type A clones S56P and S167P with an identical amino acid exchange at different positions of BSLA were investigated to obtain information about possible amino acid position effects. Furthermore, the clone K170E exemplified Type B clones. In addition to the characteristic growth parameters (Figure 7A), Figure 7B depicts a SDS-PAGE analysis of the soluble and Figure 7C of the insoluble protein fractions.

##### *E. coli* clone expressing the wild type BSLA

As presented in Figure 7A, during the first phase of the cultivation (I), the clone expressing wild type BSLA shows an initial increase in OTR and biomass signal due to growth on glucose. Its depletion corresponds with a small drop in the OTR after 3 h (first grey dotted line). In the second cultivation phase (II), further growth on glycerol occurs until 5 h accompanied by an increase in the OTR up to its first peak at 17 mmol/L/h. In this phase, no product formation is observed by fluorescence measurement. However, a small increase in the target protein content is found after 4.5 h by densitometric analysis. At the end of the second cultivation phase (second grey dotted line), the target protein production phase is initiated, and shortly after, the transition point from exponential to linear biomass formation occurs. Cultivation phase three (III) is characterized by a strong increase in product fluorescence signal and protein content with parallel consumption of the inducing compound lactose and the energy rich carbon source glycerol. As a result, the OTR decreases from its first peak to a minimum of 12 mmol/L/h after 10.5 h, and biomass growth is linear. Corresponding to the reduced growth rate, a strong increase in the plasmid copy number can be detected. Such correlation was previously described in literature [9,45,56]. At the end of the third cultivation phase (third grey dotted line), the inducing compound lactose becomes depleted, correlating with a maximum target protein content of about 31% and a maximum plasmid copy number of 182. During the fourth cultivation phase (IV), the residual glycerol is consumed leading to a small OTR increase up to a second peak of about 19 mmol/L/h. A further rise in biomass signal (scattered light intensity) up to its maximum (80 a.u.), a slight increase in the product formation, as well as a decrease in the plasmid copy number are observed. Glycerol depletion after 12 h (fourth grey dotted line) then results in a sharp drop in the OTR, a constant product fluorescence signal of 2.6 a.u. and a further decrease in the plasmid copy number. The monitored decrease in biomass formation is attributed to a change in the cell morphology [51-53]. In general, the optical measuring signals of biomass and product formation determined using the BioLector device shall only be considered during the phase of active respiration. Thereafter, factors like evaporation, cell lysis, or morphological changes can influence the measuring signals [51-53]. Because of the well-buffered medium, the pH-value decreases only slightly from 7.5 to 7.3 during the entire cultivation. Furthermore, no acetate is formed (data not shown), due to the applied medium composition and cultivation conditions.

Based on the obtained online and offline data, the cultivation can generally be divided into five characteristic phases: (I) growth on glucose, (II) growth on glycerol, (III) product formation on lactose with parallel consumption of glycerol as energy source, (IV) growth on residual glycerol, (V) depletion of all original carbon sources and end of cultivation. Thus, without laborious offline analyses, these cultivation phases can be identified *via* online monitoring of the OTR progress as function of time with the RAMOS device.

##### *E. coli* clones S56P and S167P

Fermentation of Type A clones S56P and S167P (Figure **7**A) reveals the same five characteristic cultivation phases. Whereas the first OTR peak of S167P is the same as for the clone expressing wild type BSLA, it is lower for S56P (13 mmol/L/h). With regard to the second peak, both clones reach a slightly lower maximum value. In comparison to the clone expressing wild type BSLA, the cultivation duration of S56P and S167P is somewhat longer (15 h and 14 h, respectively), while the biomass formation is reduced (maximum scattered light intensity of 73 a.u. and 66 a.u., respectively). For both clones, a higher protein formation is detected. S56P reaches a product fluorescence signal of 5 a.u., resulting in a target protein content of 45%, whereas clone S167P attains a product fluorescence signal of 7 a.u. and a target protein content of 51%. The maximum plasmid copy number for S56P and S167P is reduced to 160 and 145, respectively.

The clones S56P and S167P contain identical amino acid exchanges within BSLA sequence, but at different positions. Even though S56P expresses enzymatically inactive and S167P active BSLA (Figure 5), the fermentation progress of both clones is roughly the same. As a result, neither the position of the amino acid exchange in the protein nor the enzyme activity influence the cultivation progress.

##### *E. coli* clone K170E

The clone K170E belongs to the respiration behavior Type B. Even if the OTR progress as function of time is different compared to that of Type A, the same cultivation phases have been identified (Figure 7A). A further confirmation of the five cultivation phases for two more Type B clones is presented in the supplementary data (Additional file 6). Whereas the first cultivation phase (I) with a similar growth rate and OTR progress is comparable to those of Type A clones, the second phase (II) is much shorter. Consequently, the first OTR peak after 4.5 h reaches only 8 mmol/L/h. The biomass signal at the transition point from exponential to linear growth with a scattered light intensity of only 15 a.u. is much lower than that of the Type A clones (66–80 a.u.). No product formation was detected in this phase. Due to the shorter second cultivation phase, the third cultivation phase (III) is initiated earlier. Up to this point, not only the biomass concentration, but also the amount of consumed glycerol is lower. During the third phase (III), the OTR remains nearly constant at 8–5 mmol/L/h until 10 h, correlating with a linear increase in the biomass signal. This initiates the product formation, and an increase in the protein content. One remarkable difference between Type A and Type B clones is the consumption of glycerol during this phase. Type A clones consume a relatively high amount of glycerol in parallel with lactose, thereby leading to a low residual glycerol concentration of 1–2 g/L at the end of cultivation phase three (third grey dotted line). In contrast, the residual glycerol concentration of clone K170E is about twice as much (3 g/L) due to the higher glycerol concentration at the beginning of the this phase and the reduced parallel glycerol consumption. Accordingly, the high amount of residual glycerol causes an exponential increase in OTR (up to 31 mmol/L/h) and biomass signal (scattered light intensity, up to 92 a.u.) in the fourth cultivation phase (IV). The final product fluorescence signal is 3.9 a.u., and the maximum target protein content is 43%. The curve of the pH-value is the same as that of the other clones and no acetate formation can be detected (data not shown). Another difference between Type B clone K170E and the Type A clones is a reduced maximum copy number of 106 (Type B) compared to 145–182 (Type A) even though the curve of the plasmid copy number as a function of time is qualitatively similar. One possible explanation is that the metabolic burden is initiated at an earlier stage, thus, preventing a further increase of the plasmid copy number. Another probable explanation is an enhanced plasmid loss [48,57,58]. However, plasmid-free cells in general occur only rarely as long as the copy number remains high [58]. To evaluate whether the plasmid stability varied between Types A and B, the plasmid loss was quantified by selective plate counts for both types (data not shown). Thereby, it could be excluded that cells without plasmids overgrew cells containing plasmids.

Not only the maximum values and the progress of the plasmid copy number, but also the correlation between copy number and growth behavior have to be considered when comparing Type A and Type B clones. As already mentioned above, the third cultivation phase starts earlier for the Type B than for Type A clones. As a result, the induction of the protein expression, as well as the increase of the copy number start earlier. In general, both, recombinant protein expression as well as an increase of the copy number lead to a burden on the host’s metabolism which is indicated by a decreasing growth rate and a decreasing or constant OTR. Probably because of the reduced biomass at the beginning of the third cultivation phase, the maximum plasmid copy number is lower for K170E compared to Type A clones. With the end of the induction phase, the copy number and as result the metabolic burden decrease. Consequently, growth on residual glycerol is again possible, leading to the characteristic OTR pattern of Type B clones.

The only difference of the investigated clones consists in a single amino acid exchange within the recombinant lipase. As consequence of these exchanges, the initial growth in the second cultivation phase (II) is reduced for clones categorized in Type B. All correlations and effects on recombinant protein expression, progress of copy number, and the diverse consumption pattern of glycerol described above can be regarded as consequence of these small differences between the investigated clones.

##### SDS-PAGE analysis showing soluble and insoluble protein fractions

After investigating the characteristic growth parameters (Figure 7A), SDS-PAGE analysis of soluble (Figure 7B) and insoluble (Figure 7C) protein fractions was performed. The aim was to investigate if the ratio between soluble and insoluble protein fractions may cause the different respiration behavior of Type A and B clones. Equally for all clones, protein bands in the soluble fraction can be detected after 4.5 h. This correlates with the onset of product formation after 5 h (Figure 7A). Then, the amount of protein increased until 6 to 7.5 h and remained constant until the end of the cultivation. The SDS-PAGE analysis of the insoluble protein fraction shows traces of target protein after 6 h. Subsequently, an enormous increase in insoluble protein was observed until the end of the cultivation. These results suggest that protein expressed at the beginning of the production phase was folded correctly and remained in the soluble protein fraction. With increasing biosynthesis of the target proteins, a correct folding may increasingly be impeded and incorrectly folded proteins accumulate as inclusion bodies. As a result, the recombinant protein production can trigger various stress responses, e.g. heat-shock-like responses [24,59,60]. Inclusion bodies mainly consist of the recombinant protein, however, co-precipitation during the process of inclusion body preparation causes certain amounts of cellular protein to be included [61-63]. Beside insoluble cell proteins, this could also be an explanation for the additional protein bands shown here (Figure 7C). Besides protein overexpression, the histidine tag present in all target proteins might also promote the formation of inclusion bodies [64]. A previously described effect of the copy number on the rate of product accumulation [63] was not observed in our investigations. The four clones (Figure 7) show different maximum copy numbers but very similar protein contents in the soluble and insoluble protein fractions. Since the ratio of insoluble to soluble protein (Table 3) was roughly the same for all clones, this factor could be eliminated as a reason for the different respiration behaviors of Type A and Type B clones.

**Table 3:** **Ratio of insoluble to soluble protein fraction**

**Time**	**Wild type enzyme**	**S56P**	**S167P**	**K170E**
	**(His-LOV-BSLA)**			
**0.0 h**	0.0	0.0	0.0	0.0
**3.0 h**	0.0	0.0	0.0	0.0
**4.5 h**	0.0	0.0	0.0	0.0
**6.0 h**	1.0	1.0	0.8	1.0
**7.5 h**	3.6	1.9	2.1	2.3
**9.0 h**	4.4	4.5	3.1	3.8
**10.5 h**	4.1	4.0	4.1	4.1
**13.0 h**	4.4	4.2	4.2	4.1
**14.5 h**	3.1	3.0	3.9	3.8
**18.0 h**	3.8	3.4	3.3	3.5

Ratio of insoluble to soluble protein fraction determined from TotalLab TL100 software analyzing the relative peak area of the respective protein bands. Ratios are calculated for four *E. coli* BL21(DE3) clones presented in Figure 7.

## Conclusions

In this study, the influence of single amino acid exchanges in heterologous enzyme on protein production and metabolic activity of the respective expression host *E. coli* BL21(DE3) was investigated. Therefore, 15 *E. coli* clones expressing fusion tags, wild type lipase, or different lipase variants were compared during cultivation under non-inducing and inducing growth conditions.

As a result, no differences in respiration activity among the 15 clones were obtained under non-inducing conditions. Under inducing conditions, however, even small variations in the amino acid sequence of the target protein led to strong, highly reproducible differences in respiration activity and target protein production. A quantitative evaluation of the OTR as a function of time allowed the classification of the clones into two types of respiration behavior named Type A and Type B. With respect to the OTR curves, five characteristic cultivation phases could be identified, providing information about the time points of the depletion of the different carbon sources as well as about biomass and product formation. While phases of constant or decreasing OTR indicate strong protein production, an exponential increase in OTR occurs due to undisturbed *E. coli* cell growth. In general, clones belonging to the respiration behavior Type A were identified as clones with higher product formation, whereas clones belonging to Type B showed stronger biomass formation. Furthermore, a positive correlation between final product fluorescence signal and cultivation duration was observed.

Metabolic costs for the amino acid biosynthesis, enzyme activity, plasmid copy number, formation of inclusion bodies as well as the ratio of insoluble to soluble protein were investigated as potential factors causing the observed patterns of respiration behavior. So far, the shorter initial growth phase of Type B clones and its impact on biomass and copy number seem to have an influence. However, no particular factor could yet be identified as being exclusively responsible. Therefore, the influence of codon usage, mRNA content, as well as metabolome data are currently investigated since they might provide a deeper understanding of the underlying phenomena.

This study has proven that small variations in the gene sequence resulting in the exchange of just a single amino acid in a recombinant protein in *E. coli* influence the metabolic burden of the expression host during protein production. The two applied small-scale online monitoring systems (Respiration Activity MOnitoring Systems (RAMOS) and BioLector) allow the real-time detection of even smallest differences in respiration activity, biomass and protein production in the *E. coli* clones investigated. This study underscores the importance of parallel online monitoring systems to unveil the relevance of single amino acid exchanges for the expression of a recombinant protein.

## Methods

### Microorganism and target protein

*Escherichia coli* DH5α was used for cloning and amplification.

All cultivation experiments were conducted with *E. coli* BL21(DE3), containing the plasmid pET22b(+) (Novagen, Merck, Germany) with genes encoding different *Bacillus subtilis* lipase A (BSLA) [36] variants and also including a N-terminal His_6_ tag and a flavin-based fluorescent protein (FbFP) derived from the Light, Oxygen, Voltage (LOV) domain of the *Bacillus subtilis* YtvA photoreceptor (LOV tag) [37,38] (Table 1, Additional file 1). The amino acid exchanges within BSLA were chosen randomly and distributed over the entire amino acid sequence.

### Site directed mutagenesis

The mutations of the gene encoding BSLA were introduced by site directed mutagenesis. Polymerase chain reactions (PCR) were carried out with the modified SPRINP method of Edelheit *et al.* [65]. The amplification was carried out in two separate 25 μL reactions with each 10–50 ng of template, 0.2 pM of either the forward or reverse primer (synthetized by eurofins MWG Operon, Germany), 0.2 mM dNTPs, 3% DMSO (v/v) and 1 U of Phusion high fidelity DNA polymerase in Phusion GC-buffer containing 7.5 mM MgCl_2_ (Thermo Scientific, Germany). The PCR conditions were as follows: initial denaturation at 98°C for 10 min followed by 23 cycles of 98°C for 1 min, 55°C for 1 min and 68°C for 3.5 min followed by a final elongation step at 68°C for 7 min. The PCR was paused after 5 cycles to combine the forward and reverse primer reaction and then was continued for the remaining 18 cycles. Template DNA was removed with 30 U *Dpn*I at 37°C for 16 h. The reaction was stopped at 75°C for 15 min followed by PCR purification (Analytik Jena, Germany). An aliquot of 1 μL was transformed into *E. coli* DH5α electrocompetent cells and plated onto selective Lysogeny Broth (LB) [66] agar plates, incubated overnight at 37°C. Positive transformants were sequenced by eurofins MWG Operon (Germany) to ensure successful mutagenesis. For subsequent expression experiments, the constructed plasmids carrying the different mutations (Table 1) were isolated, transformed into competent *E. coli* BL21(DE3) cells and preserved in 15% (w/w) glycerol at −80°C.

### Growth media

#### Non-inducing media

For growth under non-inducing conditions, two different media were used. The first precultivation applied complex Terrific Broth (TB) [67] medium consisting of 12 g/L tryptone, 24 g/L yeast extract, 12.54 g/L K_2_HPO_4_, 2.3 g/L KH_2_PO_4_ and 5 g/L glycerol (all ingredients from Roth, Germany) dissolved in water. The pH-value was 7.2 ± 0.2 without adjustment. The second precultivation was carried out in modified Wilms-MOPS mineral medium according to Wilms *et al.* [68] that consisted of 5 g/L glycerol, 0.5 g/L glucose, 5 g/L (NH_4_)_2_SO_4_, 0.5 g/L NH_4_Cl, 3 g/L K_2_HPO_4_, 2 g/L Na_2_SO_4_, 41.85 g/L (N-Morpholino)-propanesulfonic acid (MOPS), 0.5 g/L MgSO_4_ · 7H_2_O, 0.01 g/L thiamine hydrochloride, 0.1 g/L ampicillin, 1 mL/L trace element solution [0.54 g/L ZnSO_4_ · 7H_2_O, 0.48 g/L CuSO_4_ · 5H_2_O, 0.3 g/L MnSO_4_ · H_2_O, 0.54 g/L CoCl_2_ · 6H_2_O, 41.76 g/L FeCl_3_ · 6H_2_O, 1.98 g/L CaCl_2_ · 2H_2_O, 33.4 g/L Na_2_EDTA (Titriplex III)]. The pH-value was adjusted to 7.5 with NaOH. All medium components were sterilized separately by autoclaving or filtration before mixing.

#### Inducing media

For growth under inducing conditions, the previously described modified Wilms-MOPS mineral medium supplemented with 2 g/L sterilized lactose as inducing compound [4-6] was used. This medium is referred to as Wilms-MOPS mineral autoinduction medium.

### Cultivations and online analysis using RAMOS and BioLector devices

An overview of the different cultivation steps, applied cultivation systems, as well as the determined online and offline data is given in Figure 1. To allow the comparability of cultivation conditions in RAMOS, separate shake flasks, and BioLector device, the shaking conditions were carefully selected to ensure unlimited growth conditions. By preventing an oxygen limitation, a negative influence on growth can be avoided. In this way, comparable cultivation conditions can be provided in all cultivations systems.

#### Precultivations

Precultivations were performed in modified 250 mL shake flasks in an in-house RAMOS device [32,33] with a filling volume of 10 mL. Commercial versions of the RAMOS device are available from Kuhner AG, Birsfelden, Switzerland or HiTec Zang GmbH, Herzogenrath, Germany. The cultures were grown at 37°C using an orbital shaker (ES-X Lab-Shaker, Kuhner AG, Switzerland) with a shaking diameter of 50 mm and a shaking frequency of 350 rpm. For the first precultivation, 10 mL of TB medium were inoculated with 100 μL from a cryoculture. As illustrated in Figure 2 by arrows, cultures were grown for 3 h and then harvested at an OTR of 40–50 mmol/L/h. For the second precultivation, 10 mL of Wilms-MOPS mineral medium were inoculated with culture broth from the first precultivation. The initial optical density (OD_600_) was set at 0.1. Cultures were grown for 5 h and were harvested at an OTR of 25–35 mmol/L/h (Figures 1 and 2).

#### Main cultivations

The main cultivations were carried out in Wilms-MOPS mineral autoinduction medium in parallel in the RAMOS device, separate shake flasks for generating samples for offline analysis, and the BioLector device (Figure 1). A master mix was inoculated with culture broth from the second precultivation with an initial OD_600_ of 0.1, and was distributed among the different cultivation systems.

#### RAMOS cultivations

The Respiration Activity MOnitoring System (RAMOS) [33] enables the online measurement of the Oxygen Transfer Rate (OTR) as an indicative parameter for growth and metabolic activity of the investigated organisms. RAMOS cultivations were carried out in modified 250 mL flasks [32] in the RAMOS device with 10 mL filling volume. Moreover, cultivations were performed at 37°C using an orbital shaker with a shaking diameter of 50 mm and a shaking frequency of 350 rpm.

#### Shake flask cultivations

Shake flask cultivations were carried out in conventional 250 mL Erlenmeyer flasks with 10 mL filling volume under identical cultivation conditions as described for the RAMOS [32,33] cultivations. The culture broth from separate shake flasks was taken at different time points of the cultivation to measure the carbon source concentrations via HPLC, the product via SDS-PAGE, pH-value and BSLA activity, as well as to determine the plasmid copy number per cell via qPCR.

#### Microtiter plate cultivations

Microtiter plate cultivations were conducted in 48-well Flowerplates (m2p-labs GmbH, Germany) in the BioLector device [34,35] which allows an online measurement of biomass and product formation per volume. Each well is irradiated with light of a defined wavelength (excitation), so that the backscattered light (indicator for biomass) or fluorescence (indicator for fluorescent products) is detected and analyzed. The filling volume was set at 1 mL. All cultivations were performed at 37°C using an orbital shaker with a shaking diameter of 3 mm and a shaking frequency of 1500 rpm.

### Offline analysis

#### Carbon sources

The concentrations of the carbon sources glucose, lactose, and glycerol were determined by HPLC (Ultimate, Dionex, Germany), equipped with an organic acid resin column (250 × 8 mm, CS Chromatographie Service, Germany). The eluent was 5 mM H_2_SO_4_ at a flow rate of 0.8 mL/min and 60°C. Peaks were detected by recording the refractive index (Shodex RI-101, Shodwa Denko Europe, Germany).

#### Recombinant protein

The recombinant protein (BSLA) was analyzed by sodium dodecylsulfate polyacrylamide gel electrophoresis (SDS-PAGE). Either total protein based on biomass was determined or soluble and insoluble fractions based on volume were measured.

For total protein, first the OD_600_ of the culture was measured. After centrifugation and removal of the supernatant, the OD_600_ was set at 5 by adding a 3:1-mixture of water and four-fold concentrated NuPAGE LDS Sample Buffer (Invitrogen, Germany). The suspension was shaken in a thermo shaker at 1000 rpm and 70°C for 10 min. For analysis, the SDS-PAGE device (Invitrogen, Germany) was equipped simultaneously with up to two gels (4–12% Bis-Tris, Invitrogen, Germany). A volume of 20 μL of the prepared samples and 15 μL of the protein marker (Roti-Mark Standard, Roth, Germany) were transferred to the gel. The running process was operated according to the manufacturer’s instructions (running time 35 min, maximum current 200 V, and maximum power 0.25 W). The gels were stained overnight in Roti-Blue staining solution (Roth, Germany) under gentle shaking at room temperature and destained with 25% methanol for 2 h. Target protein content relative to the total cellular protein was determined after electrophoresis by densitometry within the TotalLab TL100 (TotalLab Ltd, UK) software using one-dimensional gel analysis: lanes were created automatically; background was subtracted using the rolling ball method with a radius of 100; and detection of protein bands was done with a minimal slope of 100.

For determining the soluble and insoluble protein fractions, a cell pellet from 200 μL culture broth was suspended in 300 μL of BugBuster Protein Extraction Reagent (Novagen, Merck, Germany) with added DNase I (25 U/mL; Applichem, Germany) and lysozyme (1000 U/mL; Roth, Germany). The suspension was incubated at room temperature under gentle shaking for 20 min and subsequently centrifuged at 14000 rpm at 4°C for 20 min. Afterwards, the supernatant containing the soluble protein was transferred to a new reaction tube, while the pellet containing the insoluble protein was suspended in 300 μL water. Sample volumes of 60 μL of either the supernatant or the suspended pellet, were mixed with 20 μL of four-fold concentrated sample buffer and shaken in a thermo shaker at 1000 rpm and 70°C for 10 min. The gel loading (4–12% Bis-Tris, Invitrogen, Germany), the running process, as well as the gel staining were performed as described for the determination of the total protein. After electrophoresis, the ratio of insoluble to soluble protein fraction was determined using densitometry within the TotalLab TL 100 (TotalLab Ltd, UK) software. The peak areas of the respective protein bands from insoluble and soluble protein fraction referring to a standard band from protein marker (43 kDa; Roti-Mark Standard, Roth, Germany) were analyzed and the ratio of insoluble to soluble protein fraction was calculated (Table 3).

#### pH-value

The pH-value was measured using an InLab Easy pH electrode (Mettler Toledo, Germany) with a CyberScan pH 510 meter (Eutech Instruments, Thermo Scientific, Germany).

#### BSLA activity

BSLA activity was determined in cell pellets obtained from 5 mL culture broth suspended in 1 mL BugBuster Protein Extraction Reagent (Novagen, Merck Germany) with added DNase I (25 U/mL; Applichem, Germany) and lysozyme (1000 U/mL; Roth, Germany). The mixture was incubated at room temperature under gentle shaking for 20 min and centrifuged at 14000 rpm at 4°C for 20 min. BSLA activity was measured with colorless *para*-nitrophenol butyrate (*p*NPB) as the substrate. A volume of 90 μL of 50 mM potassium phosphate buffer (pH 8) and 10 μL of the supernatant from protein extraction were filled into a well of a 96-well microtiter plate and 100 μL of substrate solution composed of 2.63 μL *p*NPB, 1.5 mL acetonitrile, and 13.5 mL 50 mM potassium phosphate buffer (pH 8), was added. The reaction kinetics were monitored for 5 min through the increase in absorption at 410 nm caused by the release of yellow *para*-nitrophenolate from the substrate.

#### Plasmid copy number

The copy number of plasmids per genome copies was determined by real-time quantitative polymerase chain reaction (qPCR) [69]. Two primer sets specific to the β-lactamase gene (*bla*) and to the T7 RNA polymerase gene (*T7 RNApol*) were designed using the Primer3 web server [70]. The primer sequences are shown in Table 4 (synthetized by eurofins MWG Operon, Germany). Total DNA extraction from culture broth was performed using the QIAamp DNA Mini kit (Qiagen, Germany), following a method for bacterial cells described in the manual. The concentration of extracted DNA was quantified using a NanoDrop 2000c instrument (Thermo Scientific, Germany) and diluted to 2 ng/μL. The qPCR mixture was prepared using Power SYBR Green PCR Master Mix (Applied Biosystems, Life Technologies, Thermo Fisher Scientific, Germany). The thermal cycling protocol was performed according to manufacturer’s instructions on a Mastercycler ep realplex (Eppendorf, Germany). The thermal cycling steps were as follows: initial denaturation at 95°C for 10 min followed by 40 cycles of 15 s at 95°C and 1 min at 60°C. After the amplification, a melting curve analysis with a temperature gradient from 60–95°C was performed to confirm that only specific products were amplified. The relative quantification referring to genome copies was performed by the ΔΔC_T_ method [71].

**Table 4:** **Sequences of primers for qPCR**

**Target**	**Primers (5′ → 3′)** ^**a**^	**Length (nt)**	**Primer position**	**Product size (bp)**
***Bla***	F: AGTTCTGTCTCGGCGCGTCT	20	1331-1350	96
	R: ACTCCAGTCGCCTTCCCGTT	20	1426-1445	
***T7 RNApol***	F: AGGACTGCTTACGCTGGCGA	20	1488-1507	116
	R: TGATGCGCTCAGGGAACGGA	20	1603-1622	

^a^F and R indicate forward and reverse primers, respectively.

## Additional files

Additional file 1:
**Investigated target proteins.** (A) His_6_ tag (polyhistidin tag) fused to LOV tag (FbFP, flavin-based fluorescent protein based on the Light, Oxygen, Voltage (LOV) domain of the *Bacillus subtilis* YtvA photoreceptor), molecular weight 15 kDa. (B) His_6_ tag fused to LOV tag and wild-type lipase (BSLA, *B. subtilis* lipase A) or BSLA variants containing single amino acid exchanges, molecular weight 35 kDa. Additional file 2:
**Cultivations of** ***E. coli*** **BL21(DE3) without plasmid under non-inducing and inducing conditions.** Oxygen transfer rate (OTR) as a function of time obtained from four independent replicate experiments for: (A) first preculture performed in complex TB medium, (B) second preculture performed in Wilms-MOPS mineral medium, and (C) main culture performed in Wilms-MOPS mineral autoinduction medium. Cultivation conditions: 37°C, 250 mL flasks, filling volume 10 mL, shaking frequency 350 rpm, and shaking diameter 50 mm. Additional file 3:
**Cultivations of 15** ***E. coli*** **BL21(DE3) clones in complex autoinduction medium expressing fusion tags, wild type BSLA, or different BSLA variants as specified in Table** **1** **.** Oxygen transfer rate (OTR) as a function of time for 15 *E. coli* BL21(DE3) clones (1–8: top, 9–15: bottom) in complex autoinduction medium (commercial Overnight Express Instant TB medium, Novagen, Merck, Germany). Cultivation conditions: 37°C, 250 mL flasks, filling volume 10 mL, shaking frequency 350 rpm, and shaking diameter 50 mm. Additional file 4:
**Reproducibility of biomass formation of 15** ***E. coli*** **BL21(DE3) clones under inducing conditions expressing fusion tags, wild type BSLA, or different BSLA variants as specified in Table** **1** **.** Biomass (scattered light) as function of time obtained from 2–6 independent replicate experiments performed in Wilms-MOPS mineral autoinduction medium containing 0.5 g/L glucose, 2 g/L lactose and 5 g/L glycerol. The measured data is presented as dots, the arithmetic mean as line, and the standard deviation as colored area around the arithmetic mean. Type A clones are presented with white background, Type B clones with grey background. Cultivation conditions: 37°C, 48-well Flowerplate, filling volume 1 mL, shaking frequency 1500 rpm, shaking diameter 3 mm. Additional file 5:
**Reproducibility of product formation of 15** ***E. coli*** **BL21(DE3) clones under inducing conditions expressing fusion tags, wild type BSLA, or different BSLA variants as specified in Table** **1** **.** Product (fluorescence) as function of time obtained from 2–6 independent replicate experiments performed in Wilms-MOPS mineral autoinduction medium containing 0.5 g/L glucose, 2 g/L lactose and 5 g/L glycerol. A reference from 3 independent replicate experiments performed in non-inducing Wilms-MOPS mineral medium containing 0.5 g/L glucose and 5 g/L glycerol is presented for the clone expressing the wild type enzyme. The measured data is presented as dots, the arithmetic mean as line, and the standard deviation as colored area around the arithmetic mean. Type A clones are presented with white background, Type B clones with grey background. Cultivation conditions: 37°C, 48-well Flowerplate, filling volume 1 mL, shaking frequency 1500 rpm, shaking diameter 3 mm. Additional file 6:
**Detailed characterization of two** ***E. coli*** **BL21(DE3) clones belonging to respiration behavior Type B cultivated under inducing conditions.** Characteristic growth parameters (oxygen transfer rate, biomass formation, product formation, and carbon source concentrations) of two *E. coli* BL21(DE3) clones belonging to respiration behavior Type B (BSLA variants I12C and F17P) during the cultivation in Wilms-MOPS mineral autoinduction medium. The five cultivation phases (I-V) are specified in Figure 7. Cultivation conditions: 37°C, 250 mL flasks, filling volume 10 mL, shaking frequency 350 rpm, shaking diameter 50 mm (in RAMOS); 37°C, 48-well Flowerplate, filling volume 1 mL, shaking frequency 1500 rpm, shaking diameter 3 mm (in BioLector).

## Abbreviations

a.u.: Arbitrary unit
BSLA: *Bacillus subtilis* lipase A
*E. coli*: *Escherichia coli*
FbFP: Flavin-based fluorescent protein
HPLC: High performance liquid chromatography
IPTG: Isopropyl β-D-1-thiogalactopyranoside
LOV: Light, oxygen, voltage
OTR: Oxygen transfer rate
(q)PCR: (quantitative) polymerase chain reaction
RAMOS: Respiration activity monitoring system
SDS-PAGE: Sodium dodecylsulfate polyacrylamide gel electrophoresis
