# Supplementary figures and images for: Exchange of single amino acids at different positions of a recombinant protein affects metabolic burden in Escherichia coli
Source: Microb Cell Fact. 2015 Jan 23;14:10. doi: 10.1186/s12934-015-0191-y (PMC4307990; doi:10.1186/s12934-015-0191-y)

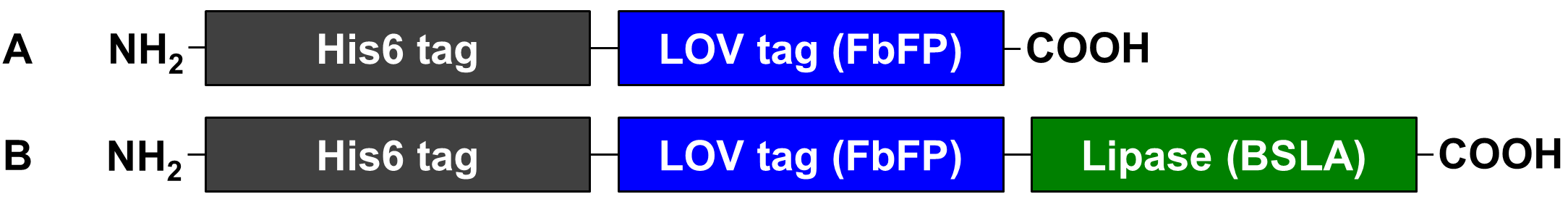

Supplement: Additional file 1: — Investigated target proteins. (A) His6 tag (polyhistidin tag) fused to LOV tag (FbFP, flavin-based fluorescent protein based on the Light, Oxygen, Voltage (LOV) domain of the Bacillus subtilis YtvA photoreceptor), molecular weight 15 kDa. (B) His6 tag fused to LOV tag and wild-type lipase (BSLA, B. subtilis lipase A) or BSLA variants containing single amino acid exchanges, molecular weight 35 kDa. [file 12934_2015_191_MOESM1_ESM.tiff]
